# Comparing online and onsite simulation modules for improving knowledge and confidence in disaster preparedness among undergraduate medical students

**DOI:** 10.1186/s12245-024-00667-5

**Published:** 2024-07-18

**Authors:** Vimal Krishnan S, Aaditya Katyal, Soumya S Nair, Kirtana Raghurama Nayak

**Affiliations:** 1 Kasturba Medical College, Manipal, Manipal Manipal Academy of Higher Education (MAHE), Manipal, Udupi, Karnataka India; 2 Centre for Resuscitation, Acute care and Simulation Training (CReST)), Manipal Academy of Higher Education (MAHE), Manipal, India

**Keywords:** Simulation, Disaster preparedness, COVID-19 pandemic, Tabletop exercise

## Abstract

**Background:**

Disaster preparedness is one of the critical strategies for effectively managing disasters and has been an area of high focus in the healthcare sector over the past few decades. The current Indian medical undergraduate curriculum does not describe any novel methods for disaster preparedness training. There is a need for a better understanding of novel teaching-learning methods and modes for administering disaster preparedness training among Indian medical students.

**Objectives:**

Describe the undergraduate medical students’ baseline knowledge and confidence level of disaster preparedness. Compare undergraduate medical students’ knowledge scores and confidence levels on disaster preparedness after online and onsite delivery of the disaster preparedness module.

**Methods:**

In this educational interventional study, 103 medical students were divided into two groups and subjected to an online or onsite session of the validated disaster preparedness module (based on the COVID-19 pandemic), encompassing a simulation-based tabletop exercise. Baseline testing was done for 52 participants in the online group and 51 in the onsite group of the study. Post-intervention, they were assessed with single-response type MCQs for knowledge and Likert scale-based questions for confidence scores. The pretest and posttest scores were collected, and the data were analysed using two-tailed t-tests for paired analysis of within-group (online group or onsite group) and heteroscedastic analysis of between-group datasets.

**Results:**

One hundred and three participants completed the exercise—52 participants were from the online group, and 51 were from the onsite group. After the intervention, there was a statistically significant increase in knowledge and confidence in both online and onsite groups. There is, however, no significant difference in the ‘percentage change’ in ‘knowledge’ or ‘confidence’ between the groups.

**Conclusions:**

Our study indicates that the disaster preparedness module, delivered online and onsite, improves knowledge and confidence among undergraduate medical students. However, there is no superiority between one mode of delivery and the other. We conclude that online training can facilitate disaster preparedness training as a corollary to the prescribed traditional training methods for undergraduate medical students in India.

**Supplementary Information:**

The online version contains supplementary material available at 10.1186/s12245-024-00667-5.

## Background

Healthcare systems all over the world are involved in disaster response [1, 2]. Disaster preparedness has been an area of high focus for multiple domains and more so for the healthcare sector over the past few decades. Healthcare professionals must be prepared for disasters, including bioterrorism events, to accept and treat victims in large numbers [3]. Disaster medicine focusing on disaster preparedness has led to multiple capacity-building programs globally. The World Health Organisation (WHO) used multiple tabletop exercise-based training strategies for pandemic preparedness and, more recently, during the COVID-19 pandemic [4, 5]. One of the major prerequisites for initiating a preparedness plan is to ensure a proper Hazard Vulnerability Analysis (HVA) before formalising a Disaster Preparedness Plan for any institute [6]. HVA would show where to focus and what resources to consider for mitigation and preparedness strategies specific to the particular institute/region [7]. A defined chain of command and a Hospital Incident Command System (HICS) must be essential for an appropriate and organised response during disasters [8].

The recent pandemic has led to an explosive growth of online resources and modes of teaching-learning and assessment methods in medical education. The complexity of the situation has been dissected well in literature [9]. Online interprofessional disaster preparedness training among healthcare professionals has been done earlier, exhibiting significant improvement in knowledge and confidence in the team [10]. There are studies showing the impact of disaster preparedness training using the online mode of delivery, even in the paediatric population [11]. A recent systematic review by Ashcroft et al. highlighted the skewing of disaster preparedness training for medical students in the Western world. Most of the studies adopted an onsite delivery, with very few having an online mode of content delivery. They have summarised a need to integrate disaster preparedness training into global medical school curricula [12]. Tabletop exercises effectively train medical students in disaster preparedness [13]. Online mode of disaster preparedness training as an elective for medical students has received positive feedback and improved confidence among medical students [14].

The Pollard et al. study objectively showed us that medical students preferred hands-on training over traditional didactics or independent learning, and the Wiesner et al. study proved that the disaster preparedness module significantly increases knowledge among medical students [15, 16].

Although the paradigm shift towards competency-based medical education (CBME) by the National Medical Commission (NMC) in India has revamped the curriculum towards an outcome-based model, innovative teaching-learning methods for disaster medicine have still not found their way into the curriculum [17]. It lacks innovative and experiential teaching-learning strategies for a complex topic like disaster management for young medical students with limited experience. A 2015 self-administered survey-based study by Singhal et al. in Udaipur, India, among medical interns emphasised the need for training to improve disaster preparedness knowledge, skills and attitudes. The lack of studies in disaster preparedness training among undergraduate medical students in our country has been explicitly described in the study [18]. Measuring gaps in achieving competency in Disaster management according to the current CBME curriculum is hugely challenging due to the scarcity of studies in this domain.

The current project aims to study whether a validated module on disaster preparedness can be used to train Indian medical students through an online delivery method compared to the traditional method.

## Methods

Ethical clearance from the Institute Ethical Committee (IEC1-398/2023) was obtained as the first step. Second-year medical students pursuing a Bachelor of Medicine or a Bachelor of Surgery (MBBS) were the target group. The target was 100 students in total, with 50 in each group.

### Setting

The study was conducted in Kasturba Medical College, Manipal. Online (via the Microsoft Teams platform) and onsite in our institute’s clinical skills lab/ Medical simulation centre.

### Study design

Educational interventional study.

### Subjects

Medical students (Year 2) from Kasturba Medical College, Manipal, who voluntarily enrolled for the simulation-based disaster training, were included in the study. (Convenience sampling was used, and the Phase II Bachelor of Medicine, Bachelor of Surgery (MBBS) students, those who consented to take part in the study (*N* = 103), were randomly divided into two groups- onsite Group I (*N* = 51) and online Group II (*N* = 52). The study’s objectives and methodology were explained to the students before obtaining consent. The sessions were conducted outside their regular clinical postings, and only students consenting to the study were included.

### Inclusion criteria

MBBS Students who voluntarily enrolled for the simulation-based disaster training were included.

### Exclusion criteria

All students who did not complete the pretest and posttest were excluded.

### Duration of the study

Six months (including data collection and analysis).

### Intervention

Online and Onsite Simulation-based disaster preparedness module. (see Supplementary File, Annexures) The topics covered in the module were disaster cycle, hazard vulnerability analysis, Incident command chain, and communication during disasters. It aligns with the competencies cited in the current CBME curriculum [17]. The online module used Microsoft Teams to deliver the session. The disaster preparedness module has been validated and published earlier [10].

### Tools

Disaster preparedness module (see Supplementary File, Annexures); Questionnaires [Knowledge- MCQs; Confidence- Likert scale] (see Supplementary File, Annexures); feedback (Likert scale based), (see Supplementary File, Annexures). The questionnaire has been validated earlier.

The knowledge was based on 15 single-response type multiple choice questions (MCQ), and the confidence was assessed with 15 questions based on a Likert scale of 1 to 5, where 1 = Not at All Confident, 2 = Slightly Confident, 3 = Confident, 4 = Very Confident, 5 = Extremely Confident.

### Detailed description of procedure/processes

A small group of facilitators (*N* = 8) underwent a 2-hour onsite and online sessions training. A single person conducted this training session- the lead investigator. They were trained to facilitate small groups of five to eight learners towards completing the different sections of the tabletop exercise-based module. After training the facilitators, a tabletop exercise-based training session was announced for medical students, which was scheduled outside the regular teaching schedule. On the allotted days, outside of the routine clinical posting, the consented participants completed the pretest questionnaire via Google Forms and joined the simulation session. The participants then participated in the online tabletop exercise or the onsite workshop. The workshop lasted 3 h and was conducted via the Microsoft Teams platform (virtually) or onsite via a traditional classroom in the medical college. The same module was used for the online and the onsite session. After the workshop, the participants were subjected to a post-workshop questionnaire and feedback form via Google Forms. A single faculty (lead author) delivered the content to all the groups and received support from the trained facilitators. After the session, the participants completed the posttest questionnaire. Each simulation-based tabletop exercise lasted 60 min. All participants were exposed to three simulation-based tabletop exercises and three interactive discussion sessions as part of the module. Each group was exposed to either the online or the onsite mode of delivery as part of the study to avoid cross-contamination. All groups were, however, given the option of cross-over after participating in the study.

### Statistical analysis

**Fig. 1:**
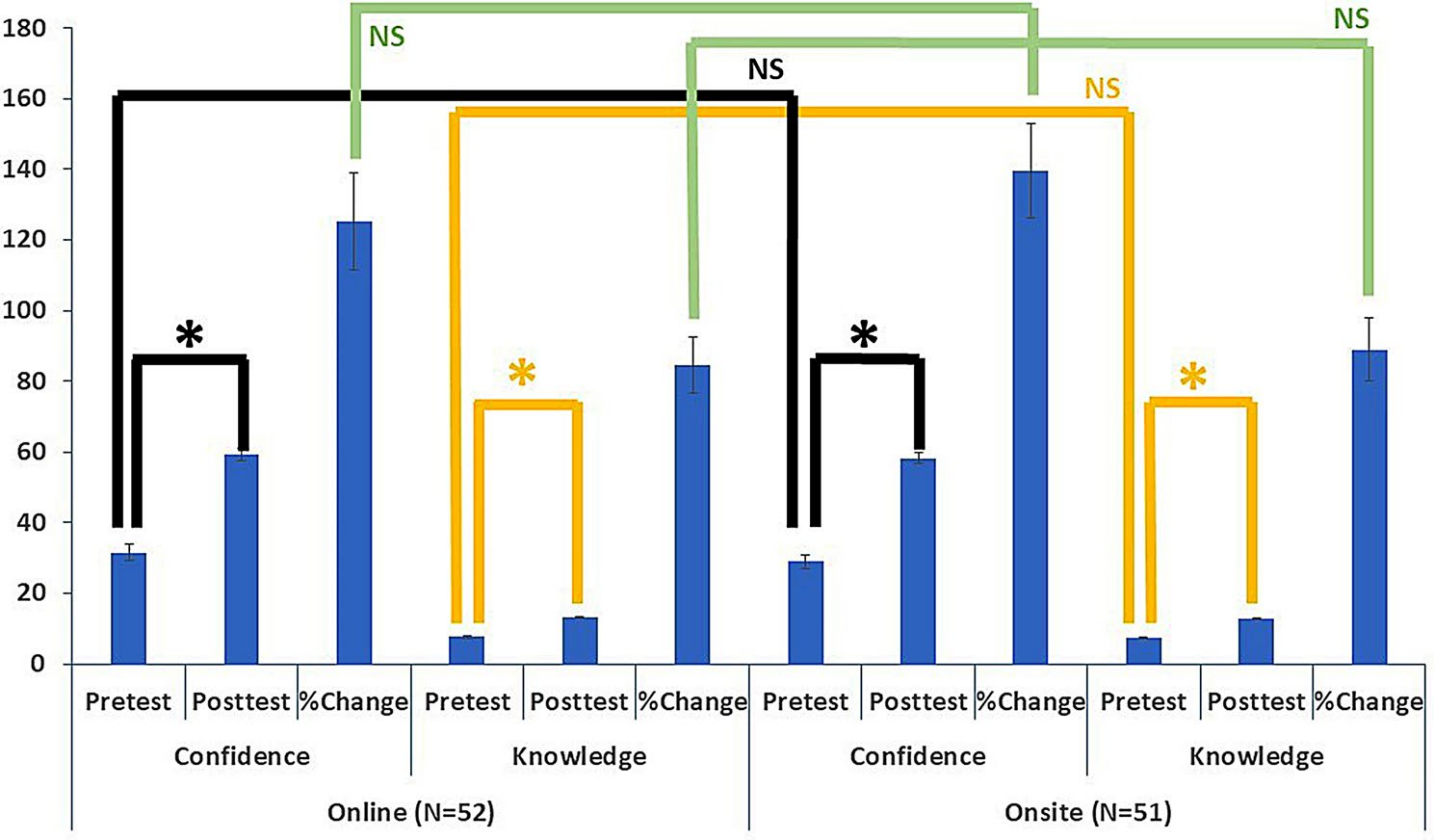
Online vs. onsite training: confidence and knowledge scores. Legend: *−Significant, NS- not significant, N- Number. Comparison of ‘confidence’ and knowledge scores from participants before and after the intervention. Evaluating the baseline by analysing the pretest scores from both online and onsite sessions (Black lines) shows no significant difference. There is no significant difference for the change in scores for ‘knowledge’ or ‘confidence’ in both modes of delivery (green line)

Statistical analysis was performed using Microsoft Excel^®^. The knowledge part was calculated and scored using a single response type MCQ, while the confidence and feedback were captured on a Likert scale. The mean, standard error of mean, and standard deviation were calculated for each group. Two-tailed t-tests were applied for paired analysis of within-group (online group or onsite group) and heteroscedastic analysis of between-group datasets (See Fig. 1; Tables 1, 2 and 3).

## Results

The participants who have undergone six exposures and completed the pretest and posttest have been incorporated for the analysis.

One hundred three participants completed the exercise—52 were from the online group, and 51 were from the onsite group. All the students who attended were from phase II of the MBBS course for our institute.

The mean, standard error of mean and standard deviation for the knowledge and confidence scores of participants from the online and the onsite group before and after the exercise were calculated (Tables 1 and 2). The changes in both groups were also calculated separately and presented in the table and figure below. Post training, there is a statistically highly significant (p<<<0.01) increase in both the knowledge and confidence in both online and onsite groups. The percentage change in scores for knowledge and confidence, compared between online vs. onsite groups, did not have a statistically significant difference (*p* > 0.05). (see Fig. 1; Table 3).

**Table 1:** Confidence and knowledge with online sessions

	Online (*N* = 52)					
	Confidence			Knowledge		
	Pretest	Posttest	%Change	Pretest	Posttest	%Change
Mean	31.61538	59.5	125.302	7.673077	13.13462	84.52783
SEM	2.203308	1.7223357	13.8692	0.30422	0.280095	8.054756
SD	15.88828	12.41994	100.0122	2.193763	2.019793	58.08367
N	52	52	52	52	52	52

Legend- SEM- Standard error of mean, SD- Standard deviation, N-number

**Table 2:** Confidence and knowledge with onsite sessions

	Onsite (*N* = 51)					
	Confidence			Knowledge		
	Pretest	Posttest	%Change	Pretest	Posttest	%Change
**Mean**	28.94118	58.31373	139.5738	7.45098	12.94118	88.97533
**SEM**	2.06358	1.462871	13.40115	0.298117	0.206581	8.797765
**SD**	14.73691	10.44699	95.70335	2.128978	1.475287	62.82861
**N**	51	51	51	51	51	51

Legend- SEM- Standard error of mean, SD- Standard deviation, N-number

**Table 3:** T-test results

T-tests were conducted across different groups and tabulated		
T-test	*P* value	Statistical significance
Online group: pre-post confidence	p<<<0.01	Highly significant
Online group: pre-post knowledge	p<<<0.01	Highly significant
Onsite group: pre-post confidence	p<<<0.01	Highly significant
Onsite group: pre-post knowledge	p<<<0.01	Highly significant
Pretest Confidence: Online vs. Onsite	0.3	Not significant
Posttest Confidence: Online vs. Onsite	0.6	Not significant
Percent change Confidence: Online vs. Onsite	0.4	Not significant
Pretest Knowledge: Online vs. Onsite	0.6	Not significant
Posttest Knowledge: Online vs. Onsite	0.5	Not significant
Percent change Knowledge: Online vs. Onsite	0.7	Not significant

## Discussion

Disaster preparedness and risk reduction have been studied extensively over the past few decades. The coronavirus disease (COVID-19) pandemic has led to an explosive increase in disaster preparedness strategies studied globally. Besides conventional teaching-learning strategies, innovative simulation-based strategies and technology integration have increased interest in disaster preparedness training.

Tabletop exercises on biological disasters for healthcare learners have significantly improved their knowledge and confidence in emergency preparedness [19]. The CBME curriculum adopted by the National Medical Commission (NMC), India, suggests the traditional teaching-learning methods of lectures or small group discussions for teaching disaster preparedness among undergraduate medical students in India [20]. Exploring innovative teaching-learning methods and comparing their effectiveness was the ultimate goal of our study. We compared two delivery modes of a validated simulation-based tabletop exercise module on disaster preparedness. Medical students from our institute are actively engaged in either online or traditional onsite strategies. Learners in both groups appreciated the innovative simulation exercise integrating a tabletop exercise, which was captured in the feedback. Bridging the gap between enthusiasm and translatory support during disasters was the intent of training undergraduate medical students in disaster preparedness. The explosion of online training after the COVID-19 pandemic has highlighted that the costs and resources needed are significantly lower than those for onsite training. The online training strategy has earlier trained undergraduate medical students in disaster preparedness as an elective subject [14]. Participants have a statistically significant increase in knowledge and confidence in disaster preparedness after implementing the module at our centre. This is expected and is similar to other studies assessing knowledge and confidence following simulation-based training among learners from the healthcare domain [21]. The innovative use of tabletop exercises that make the session learner-centric also aligns with the current wave of learner-led instruction with the teacher as a facilitator.

Interestingly, the percentage change in knowledge and confidence among learners from the online and onsite groups are similar (no statistically significant difference) (Tables 1 and 2; Fig. 1) despite the statistically significant difference noted independently in each group. This suggests that online training can be equally effective for improving knowledge and confidence in disaster preparedness among medical students compared to the traditional classroom or onsite mode. This was similar to the 2018 study comparing online and onsite training models for disaster preparedness, which revealed no significant difference between the course delivery models for knowledge gained among learners [22]. The learners from that study were a heterogeneous group, with a majority being firefighters and nurses and a smaller number of physicians than in our study of uniform learners. Although the COVID-19 pandemic paved the way for an array of online training programs globally, a comparison with the traditional method of content delivery for disaster preparedness among medical students in India has not been undertaken to date. The study’s implication will be to help educators decide on the teaching-learning strategy and incorporate the suggestions into the undergraduate curriculum. This may help reduce the gaps in disaster preparedness training for the Indian Medical Graduates in the current CBME curriculum. The study’s main limitation is that it’s done in a single centre with a small sample size. The study must be implemented at multiple sites with a larger sample size to avoid bias.

## Conclusion

The present study builds on the previously published one, focussing on online disaster preparedness training using simulation tabletop exercises [10]. Our study evaluated the effectiveness of different modes of delivering a simulation-based module- online vs. onsite. Our study indicates no significant difference in the percentage change in knowledge and confidence between the online and the onsite group. This study can catalyse the use of innovative teaching-learning methods for content delivery in the domain of disaster preparedness among undergraduate medical students.

### Electronic supplementary material

Below is the link to the electronic supplementary material.

Supplementary Material 1

## Data Availability

No datasets were generated or analysed during the current study.
