# Supplementary material for: Comparing online and onsite simulation modules for improving knowledge and confidence in disaster preparedness among undergraduate medical students
Source: Int J Emerg Med. 2024 Jul 18;17:93. doi: 10.1186/s12245-024-00667-5 (PMC11256551; doi:10.1186/s12245-024-00667-5)
Supplement: Supplementary file 1 — Supplementary Material 1 [file 12245_2024_667_MOESM1_ESM.docx]

**Annexure: SIMTEx Module**

**SIMTEx Module**

**(Simulation Tabletop Exercise for Disaster Preparedness)**

**Table of Contents**

Hospital Model Descriptions…………………………Page 3-4
Hazard Assessment Tool ………………………...…Page 5-8

Disaster Plan…………………………………............Page 9-11


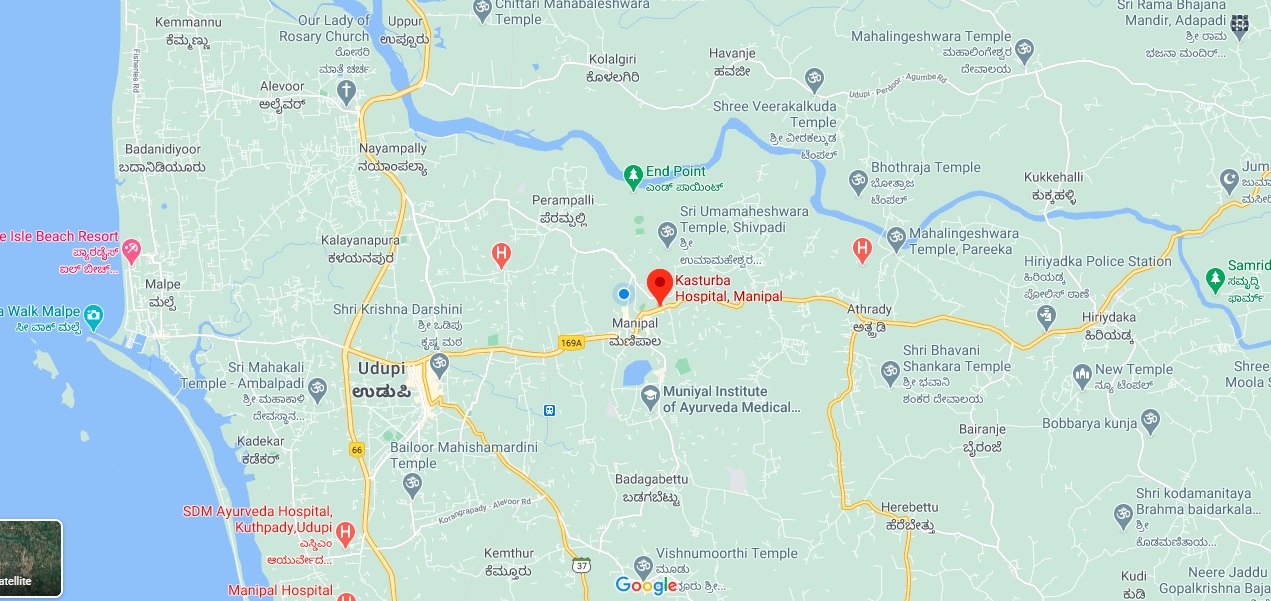


Pic courtesy- GoogleMap

**KH, Manipal, Udupi**

**Location:** refer map

**Population-** 11,77,908 (2011)

**Demographics:** 49% male, 8% <6 years old, 83% literate

**Physical Infrastructure:** to update on the bed status after surging fo COVID pandemic (discuss with MS and team)

**Industries:** clay roof tiles industry, Cashew nut processing industry, Coconut oil mills, aquaculture, thermal power, tourism, trade and banking

**Nearby Resources:** Large seaport (66km), Large airport (65km), 7 rivers, 196 vented dams

Drinking water- Swarna river- Baje Dam

Thermal power plant- 1200MW

**Recent Disasters**: Floods and cyclones, epidemics, food poisoning, accidents, bomb blasts, drought, building and fire disaster (refer page7,8)

**HAZARD ASSESSMENT**

**TTX 1- Hazard Vulnerability Asessment (HVA)**

| **HAZARD AND VULNERABILITY ASSESSMENT TOOL** | | | | | |
| --- | --- | --- | --- | --- | --- |
| **EVENT** | **PROBABILITY**  (Chance of happening) | **HUMAN IMPACT**  (Death & Injury) | **ECONOMIC IMPACT**  (Damages, business disruption) | **PREPAREDNESS** | **RISK** |
| **SCORE** | 0=N/A  1= Low  2=Moderate  3=High | 0=N/A  1= Low  2=Moderate  3=High | 0=N/A  1= Low  2=Moderate  3=High | 0=N/A  1= High  2=Moderate  3=Low | Add scores |
| **Infectious Outbreak** |  |  |  |  |  |
| **Extreme Weather** |  |  |  |  |  |
| **Terrorism/ Civil Unrest** |  |  |  |  |  |
| **Radiologic Exposure** |  |  |  |  |  |
| **Chemical/ Hazmat Exposure** |  |  |  |  |  |


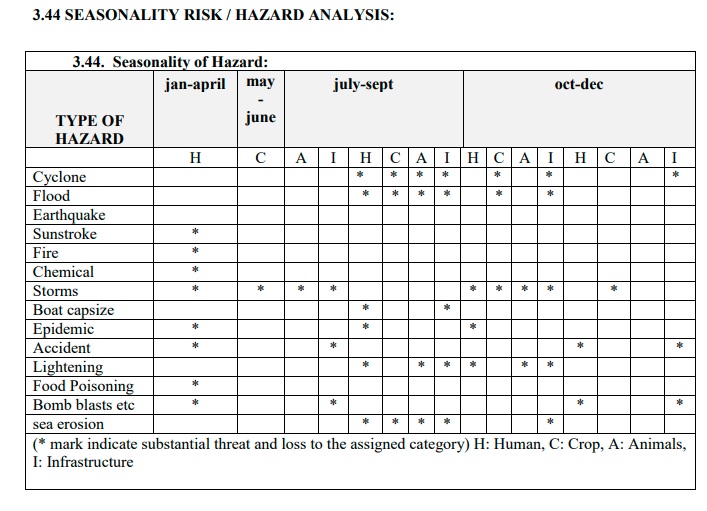


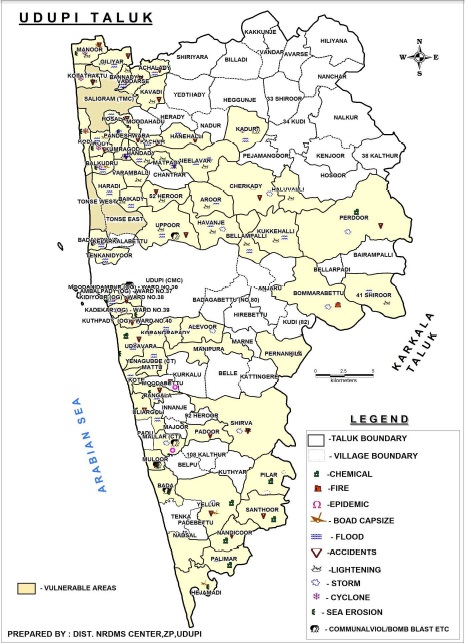


**DISASTER PLAN**

**Disaster Plan Outline:**

**Incident Command:**

**Location- ________________________________________________________**

**Staff Roles:**

**Incident Commander: ______________________________________________________________**

**Operations Officer: ______________________________________________________________**

**Safety Officer: _______________________________________________________________**

**Information Officer: _____________________________________________________________**

**Liaison Officer: ______________________________________________________________**

**TTX 2- Hospital Preparedness**

1. Describe the threat to the Hospital/ Healthcare system?
2. What are the medical issues you might face?
3. What are the safety/ security issues you might face?
4. What are the logistics(operations) issues you might face?

Ref

<https://www.cdc.gov/coronavirus/2019-ncov/downloads/HCW_Checklist_508.pdf>

<https://covid.aiims.edu/personal-protective-equipment-covid-19-preparedness/>

<https://www.mohfw.gov.in/pdf/National%20Guidelines%20for%20IPC%20in%20HCF%20-%20final%281%29.pdf>

<https://www.mohfw.gov.in/>

**TTX 3**- **Incident Response (Action plan) & communication**

1. How will you mitigate the risk of infection for your front line health care workers?
2. How will internal staff contacts of infected health workers be managed, including anticipating a shortage of available health workers, and a safe return to work strategy for infected health workers?
3. How will you address the media?

Ref

(The Management of Infected Health Workers document, also included in this document are screening considerations that participants may want to consider in their local contexts –

<https://www.who.int/publications/i/item/10665-336265> )

<https://www.mohfw.gov.in/pdf/National%20Guidelines%20for%20IPC%20in%20HCF%20-%20final%281%29.pdf>

<https://ncdc.gov.in/WriteReadData/l892s/63948609501585568987.pdf>

<https://apps.who.int/iris/bitstream/handle/10665/331499/WHO-2019-nCoV-IPC_WASH-2020.2-eng.pdf>

**Annexure: Questionnaire**

*Your feedback is valuable and will be de-identified and used for research purposes only after your consent.*

Roll No:

Age: Gender: Residence (Specify Place): Email ID:

Were you previously involved with Disaster Management- Yes/No

If Yes, Please Specify (Brief Description) ------

Have you ever attended any Disaster Preparedness Training Workshops- Yes/No

If Yes, Please Specify (Brief Description) ------

Have you even been a part of a Tabletop Training Exercise- Yes/No

If Yes, Please Specify (Brief Description) ------

**Section A**

| 1. **The following questions assess your confidence on the topic**   *Please complete the following questionnaire on a scale of* ***1-5:***  **1=Not at All Confident 2=Slightly Confident**  **3=Confident 4=Very Confident 5=Extremely Confident**  *Please circle your response to each statement.* | Not at all Confident | Slightly Confident | Confident | Very Confident | Extremely Confident |
| --- | --- | --- | --- | --- | --- |
| **Kindly ensure that all questions are answered:** | | | | |  |
| 1. Are you confident in your understanding of the Disaster Cycle? | **1** | **2** | **3** | **4** | **5** |
| 1. How confident are you on the general topic- Disaster Preparedness? | **1** | **2** | **3** | **4** | **5** |
| 1. Are you confident in activating the disaster response plan in your department? | **1** | **2** | **3** | **4** | **5** |
| 1. Are you confident of working as a team member for Disaster Preparedness planning in your hospital? | **1** | **2** | **3** | **4** | **5** |
| 1. Are you confident with the concept of Surge Capacity during disaster? | **1** | **2** | **3** | **4** | **5** |
| 1. How confident are you with your understanding of Hazard Vulnerability Analysis (HVA) in Disaster Planning | **1** | **2** | **3** | **4** | **5** |
| 1. Are you confident of doing a Hazard Vulnerability Analysis (HVA) for your hospital? | **1** | **2** | **3** | **4** | **5** |
| 1. How Confident are you in incorporating the HVA for your hospital's Disaster Preparedness plans? | **1** | **2** | **3** | **4** | **5** |
| 1. How confident are you in understanding of the Incident Command Chain structure? | **1** | **2** | **3** | **4** | **5** |
| 1. How confident are you in preparing an action plan as part of disaster preparedness? | **1** | **2** | **3** | **4** | **5** |
| 1. How confident are you in communicating with your team in a timely, well-coordinated and effective manner during a disaster? | **1** | **2** | **3** | **4** | **5** |
| 1. How confident are you in your understanding of modes of communication during a disaster? | **1** | **2** | **3** | **4** | **5** |
| 1. Are you confident of fulfilling your role in the Hospital Incident Command System (HICS)? | **1** | **2** | **3** | **4** | **5** |
| 1. Are you confident to plan for a media response during/after a disaster? | **1** | **2** | **3** | **4** | **5** |
| 1. Are you confident that a virtual tabletop exercise can help in Disaster Preparedness training? | **1** | **2** | **3** | **4** | **5** |

**Section B**

1. Which of the following is not a part of the Disaster Cycle?
2. Preparation
3. Mitigation
4. Response
5. Recovery
6. Resilience
7. Hazard Vulnerability Analysis is a strategy used for Disaster Preparedness?
8. True
9. False
10. Preparation is the first phase of a disaster
11. True
12. False
13. Which of the members are not a part of the Hospital Incident Command Chain?
14. Liaison officer
15. Public Information officer
16. Security Officer
17. Medical Superintendent
18. Receptionist
19. Disaster cycle has how many phases?
20. 1
21. 2
22. 3
23. 4
24. 6
25. Setting up of NDMA and the creation of an enabling environment for institutional mechanisms at the State and District levels is mandated by the
26. Disaster Management Act 2005
27. Sendai Framework for Disaster Risk Reduction – SFDRR
28. United Nations Framework UNFCC
29. WHO guidelines
30. HVA takes into account
31. Probability of the event
32. Economic impact of the event
33. Preparedness
34. A&B
35. All
36. Which of the following is not considered a basic component of Surge Capacity system?
37. Staff
38. Stuff
39. Safety
40. Structure
41. Which is not a part of Hospital Incident Command System
42. Operations Section
43. Finance Section
44. Logistics Section
45. Planning Section
46. None
47. The nodal ministry in charge of matters pertaining to Disaster Management
48. Ministry of Defence
49. Ministry of Home Affairs
50. Ministry of Health
51. Ministry of Urban Development.
52. Tabletop Exercises and Operations Based Exercises are the same?
53. True
54. False
55. Drills are a part of Operations Based Exercise?
56. True
57. False
58. Which is the weakest/most vulnerable link in Disaster Plan from the options given below?
59. Interdepartmental Planning
60. Resource Management
61. Communication
62. Command structure
63. Safety and Security
64. Surge Capacity and Surge Capability are the same for hospital based disasters?
65. True
66. False
67. Communications during hospital based disasters are categorised as convergent and divergent?
68. True
69. False

**Annexure: Feedback**

**Participants' Feedback**

*Your feedback will assist us to maintain and improve the quality and relevance of future simulation exercises.*

| **1. SIMULATION OBJECTIVES**  *Please circle your response to each statement.* | I Strongly Disagree | I Disagree | I Agree | I Strongly Agree |
| --- | --- | --- | --- | --- |
| **Were the following simulation objectives achieved:** | | | | |
| 1. Understand the principle of Emergency Preparedness in the disaster cycle | 1 | 2 | 3 | 4 |
| 1. Understand the Incident Command System (ICS) | 1 | 2 | 3 | 4 |
| 1. Activation and functioning of Hospital ICS | 1 | 2 | 3 | 4 |
| 1. Understand what is Hazard Vulnerability Analysis (HVA) | 1 | 2 | 3 | 4 |
| 1. Conduct a HVA for your hospital | 1 | 2 | 3 | 4 |
| 1. Role allocation in Incident Command Chain | 1 | 2 | 3 | 4 |
| 1. Preparing an action plan for the simulated scenario. | 1 | 2 | 3 | 4 |
| 1. Improve interpersonal communication to enable a timely, well-coordinated and effective response. | 1 | 2 | 3 | 4 |
| 1. Work with colleagues as a team | 1 | 2 | 3 | 4 |
| 1. Review media communications. | 1 | 2 | 3 | 4 |
| **2. SELF PREPAREDNESS**  *Please circle your response to each statement.* |  |  |  |  |
| 1. I'm more familiar with HVA and its importance in Disaster Preparedness planning for the hospital | 1 | 2 | 3 | 4 |
| 1. I'm better prepared to act and respond appropriately according to my role | 1 | 2 | 3 | 4 |
| **3. SIMULATION FACILITATION**  *Please circle your response to each statement.* |  |  |  |  |
| 1. The simulation scenario covered what I expected it to cover. | 1 | 2 | 3 | 4 |
| 1. The Facilitation helped in improving the preparedness plan. | 1 | 2 | 3 | 4 |
| 1. The virtual platform set up was appropriate. (if attended virtually) | 1 | 2 | 3 | 4 |

1. **PLEASE CHOOSE YOUR RESPONSE TO THE FOLLOWING STATEMENTS**

| 1. The duration of the simulation was right for me: | □ Yes | □ No, too long | □ No, too short |
| --- | --- | --- | --- |
| 1. The pace of the simulation was right for me: | □ Yes | □ No, too fast | □ No, too slow |

1. **HAVE YOU ANY SUGGESTIONS ABOUT HOW THIS SIMULATION COULD BE IMPROVED?**

|  |
| --- |
|  |
|  |
|  |
|  |

**Annexure: Session photos**

**
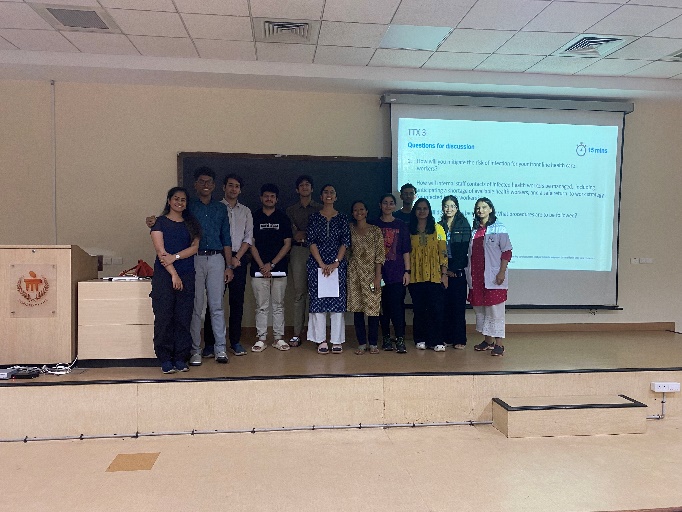

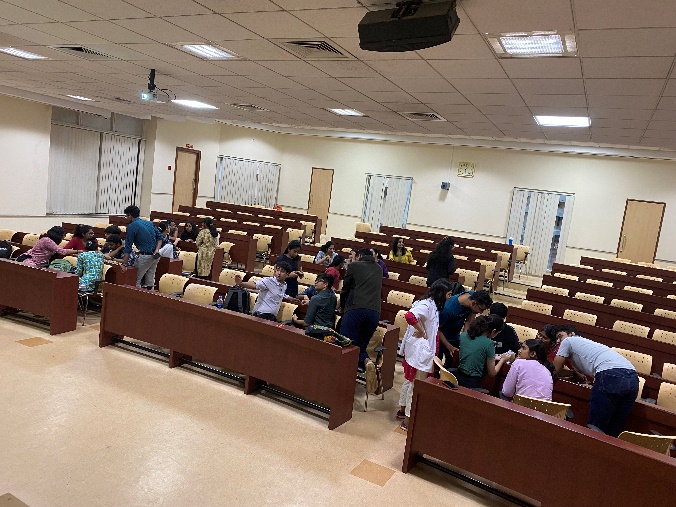
**


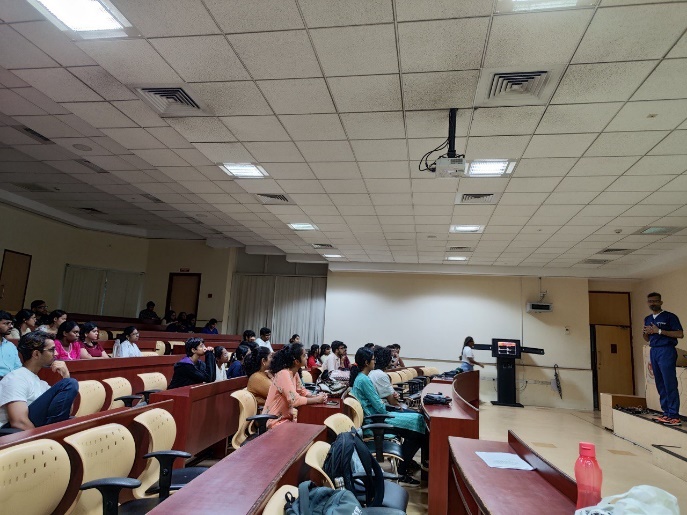

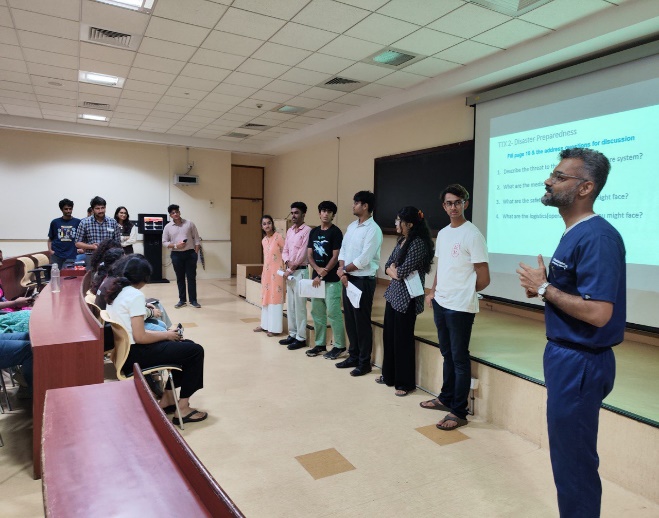


**
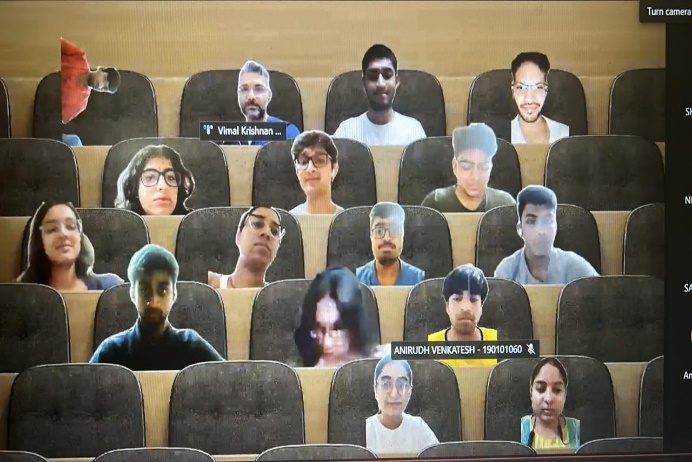

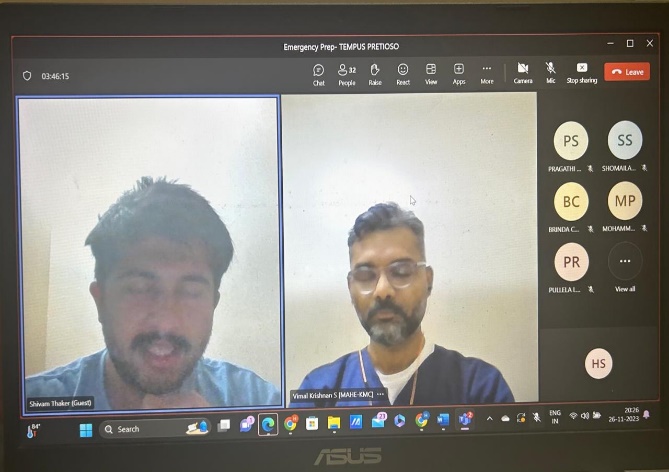
**

**Annexure 4:** photos from SIMTEx session- online and onsite sessions.
